# Case Report: Adrenocortical Oncocytoma in a Patient with a Previous Contralateral Adrenalectomy for a Cortisol-Secreting Adenoma

**DOI:** 10.3389/fsurg.2022.897967

**Published:** 2022-05-19

**Authors:** Letizia Canu, Giuliano Perigli, Benedetta Badii, Raffaella Santi, Gabriella Nesi, Silvia Pradella, Mario Maggi, Alessandro Peri

**Affiliations:** ^1^ Endocrinology, Department of Experimental and Clinical Biomedical Sciences ‘Mario Serio’, University of Florence, Florence, Italy; ^2^ Endocrine Surgery Unit, Department of Experimental and Clinical Medicine, University of Florence, Florence, Italy; ^3^ Division of Pathological Anatomy, Department of Health Sciences, University of Florence, Florence, Italy; ^4^ Department of Emergency Radiology, University Hospital Careggi, Florence, Italy; ^5^ Pituitary Diseases and Sodium Alterations Unit, University Hospital Careggi, Florence, Italy

**Keywords:** adrenal oncocytoma, adrenalectomy, adrenal-sparing surgery, Cushing syndrome, adrenal tumor

## Abstract

**Background:**

Oncocytomas are uncommon benign tumors that arise in various organs and are predominantly composed of oncocytes. Adrenocortical oncocytomas are extremely rare and are generally non-functioning.

**Methods:**

We report the case of a 40-year-old patient with a progressively enlarging left adrenal mass. At the age of 19 he had undergone right adrenalectomy for a cortisol-secreting adenoma. Radiologic features were not typical of an adenoma and positive uptake was detected at ^18^F-FDG-PET. Because of the uncertain nature of the growing lesion, it was decided to proceed to surgical resection.

**Results:**

The surgeon managed to remove the left adrenal mass, sparing the normal adrenal gland, and histology was consistent with adrenocortical oncocytoma. Corticosteroid supplementation was prescribed, but at reassessment, adrenal function was found to be preserved and treatment withdrawn.

**Conclusions:**

Adrenal oncocytoma is a rare diagnosis, but should be considered in the presence of a growing mass with non-specific radiologic appearance.

## Introduction

Oncocytomas are uncommon benign tumors arising in various organs such as the kidneys, salivary glands, thyroid, parathyroids and pituitary (1). They are predominantly composed of oncocytes, which are large polygonal cells with granular eosinophilic cytoplasm packed with mitochondria (2, 3).

Adrenocortical oncocytomas are exceedingly rare (4, 5). They almost exclusively occur in adults (6), predominantly in females (2.5:1) and in the left adrenal (3.5:1) (2). No genetic or environmental risk factors are known (5). Adrenocortical oncocytomas are generally non-functioning (up to 70% of cases), but in a significant minority of cases they may be symptomatic due to steroid production, resulting in Cushing syndrome, virilization or feminization. In most cases adrenocortical oncocytomas are discovered incidentally and the biochemical and imaging assessment is carried out according to current guidelines (7). Surgery is considered the treatment of choice (8). Total adrenalectomy is usually performed, in agreement with the large majority of interventions for adrenal lesions. Adrenal sparing procedures can be considered in selected cases, in order to prevent the risk of Addison’s disease.

We describe the case of a patient with a previous history of cortisol-secreting adrenocortical adenoma, referred to our clinic after the discovery of a contralateral lesion.

## Case Presentation

**Figure 1:**
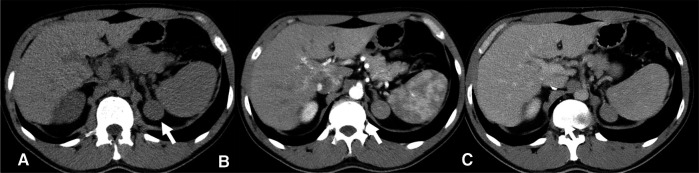
**CT scan 2018-** Homogeneous left adrenal mass with regular margins, (arrow), diameter of about 3 cm and a non-contrast CT density greater than 10 HU (**A**) not indicative of the presence of lipids. In the arterial (**B**) and venous (**C**) contrast phases, the mass presents a slightly heterogeneous appearance.

**Figure 2:**
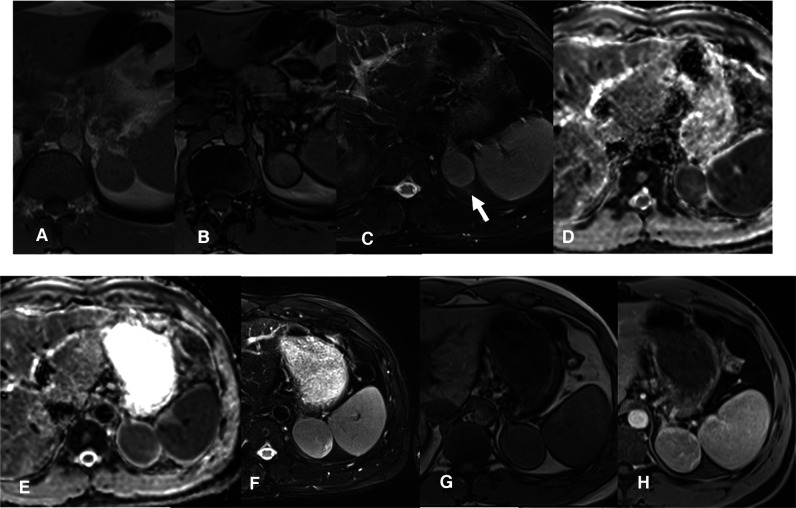
MRI scan 2019- Follow-up confirms the presence of a slightly enlarged, compared to the CT control, left adrenal solid mass. The T1 in-phase (IP) and out-of-phase (OOP) sequences (**A,B**) confirm a substantial absence of lipid content. The mass is rather homogeneous, isointense in T2 STIR (**C**, arrow), solid with restriction in the diffusion map (**D**). MRI scan 2020- A clear enlargement of the adrenal mass is present (maximum diameter about 5 cm), which is heterogeneously solid in the DWI map (**E**). Also in T2 w (**F**) the mass is larger and heterogeneous, in T1 w the mass has a parenchymal signal (**G**) with a slightly inhomogeneous post-contrast impregnation (**H**, venous phase).

A 40-year-old man was seen at our clinic in summer 2020 for a left adrenal mass. At the age of 19, he had suffered from ACTH-independent Cushing syndrome, when a right adrenal lesion was detected at CT scan, leading to adrenalectomy. Histopathology was indicative of adrenocortical adenoma. Cortisol normalization occurred after surgery and follow-up visits were scheduled, with regular assessment of adrenal function and abdomen ultrasound imaging. In 2018, a CT scan had revealed a left adrenal mass with a maximum diameter of 3 cm (Figure 1). A diagnosis of adrenocortical adenoma with low lipid content was suggested. The absence of a previous CT scan to make a comparison, the size of the mass at that time (less than 4 cm), the mass homogeneity (absence of calcifications and hemorrhagic areas), the density (30–40 HU on average, below the limit of 43 HU), the regularity of the margins and the lack of a CT delayed phase (to evaluate the wash-out) had probably orientated the diagnosis. Hormonal evaluation disclosed normal adrenal function [ACTH 29 pg/mL, serum cortisol 450 nmol/L, serum cortisol after 1 mg dexhametasone 41 nmol/L, UFC 58 mcg/24 h, renin 7.8 µU/mL, serum aldosterone 100 pg/mL, aldosterone (ng/dL)/renin (µU/mL) 1.28, urine metanephrine 25.5 µg/24 h, urine normetamephrine 105.8 µg/24 h]. At an MRI scan performed in 2019, the lesion showed a minimal size increase, and a wait-and-see strategy was decided (Figures 2A–D). Qualitative analysis of axial MRI images in and opposed-phase (Figures 2A,B) revealed no signal drop inside the lesion. Retrospective quantitative analysis with ASII showed a 7.24% reduction in signal, which does not meet the criteria for adenoma, in T2 MRI images with fat suppression the lesion was isointense. Overall, MRI features were not typical for an adenoma (9). Probably the homogeneity of the lesion, the minimal growth and the previous adenalectomy explain the ‘wait-and-see’ strategy.

**Figure 3:**
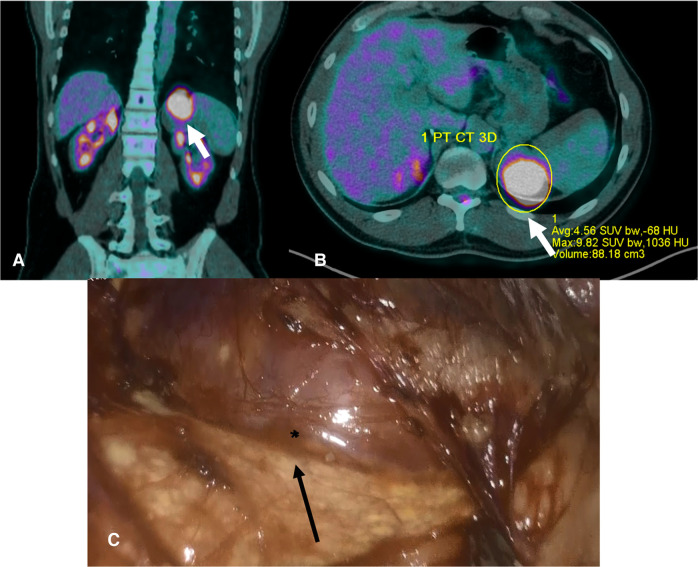
(**A,B**). PET scan 2020- The images show a well-defined lesion (arrow) involving the left adrenal region that has a SUV average of 4.56 with an average -68bHU; SUV average is higher than the liver SUV, thus suggesting a malignant lesion. (**C**). Intraoperative picture. The normal adrenal gland is indicated by the arrow, whereas the surface of the oncocytoma by the asterisk.

**Figure 4:**
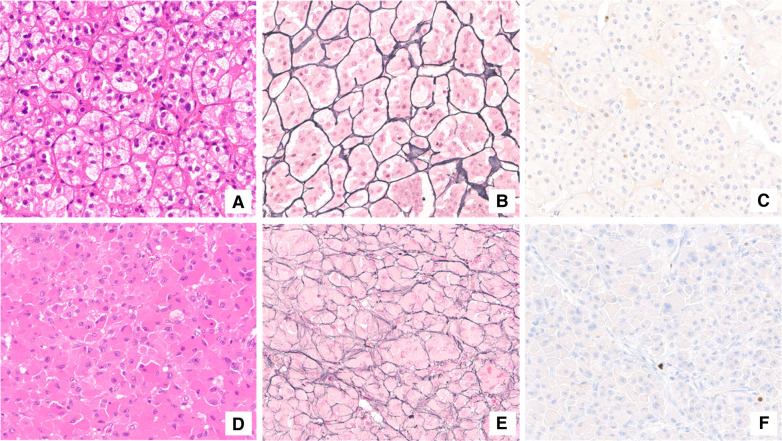
Histological assessment. Adrenocortical adenoma (**A**–**C**). The neoplasm was mainly composed of lipid-laden cells resembling those of normal zona fasciculata (**A**). The reticulin framework completely surrounded adrenal adenoma cells in small nests (**B**). Proliferation index (Ki-67) was lower than 1% (**C**). Adrenocortical oncocytoma (**D**–**F**). Oncocytic cells were characterized by granular eosinophilic cytoplasm and bland-appearing nuclei (**D**). The solid-alveolar pattern was better appreciated with reticulin stain (**E**). Ki-67 immunostaining labeled a very low number of neoplastic cells (**F**).

When we first met the patient, in the summer of 2020, a further MRI indicated disease progression, with the mass measuring 5.5 cm in greatest diameter and presence of inhomogeneities in the T1, T2 and post contrast sequences (Figures 2E–H). The lesion exhibited uptake at ^18^F-FDG-PET scan, with a SUVmax adrenal/SUVmax liver ratio of 3.5 (Figures 3A,B). At a multidisciplinary team meeting involving specialists in endocrinology, surgery and radiology, it was agreed to proceed to surgical resection. The surgeon managed to laparoscopically remove the mass, adopting a conventional antero-lateral approach to the abdominal cavity, using only three trocars (optical: in subcostal anterior axillary line, right-hand: subcostal, 5 cm laterally from the optical one, left-hand: subcostal, 5 cm medially from the optical one) with an energy-based device for dissection and clips for major vessels. The normal adrenal gland, which was completely separated from the lesion was entirely spared (Figure 3C). Histology was indicative of an adrenocortical oncocytic neoplasm and, using the Lin-Weiss-Bisceglia criteria, the tumor was categorized as benign. The revision of the histological sections obtained at the time of right adrenal surgery confirmed the previous diagnosis of adrenal adenoma (Figure 4). The post-operative course was uneventful, and blood pressure, glycemia, serum sodium and potassium were within the normal range. As a precaution, the patient was discharged with corticosteroid supplementation (oral cortone acetate 18.75 + 6.25 mg/day). After two months, adrenal function was reassessed and found to be normal (ACTH 27.1 pg/mL, basal cortisol 283.2 nmol/L, renin 35 µU/mL, aldosterone 62.1 pg/mL, ACTH stimulated cortisol 508 nmol/L). Cortone acetate supplementation was subsequently discontinued. The patient attends regular follow-up and is currently (i.e., one year after surgery) in good health.

## Discussion

Since the first description by Kakimoto et al. in 1986 (10), less than 200 cases of adrenocortical oncocytic lesions have been reported in the literature. These tumors include adrenocortical oncocytoma, borderline oncocytic neoplasm and oncocityc adrenocortical carcinoma (ACC). Traditionally, malignant behavior of adrenocortical oncocytic lesions has been thought to be rare, but at least 20% of the reported cases demonstrated borderline malignant potential or were frankly malignant (11), with a propensity for hematogenous spread (bones, liver and lungs) (12).

Adrenocortical oncocytic lesions are generally nonfunctional and detected incidentally (13), as in our patient. Hormone excess (usually cortisol, androgens or estrogens) occurs in no more than 30% of cases (13), and approximately 10% of the secreting tumors cause subclinical forms.

At CT scan or MRI, about 30% of oncocytomas may display a central fibrous scar, resulting in the so-called ‘spoke wheel’ pattern (14), not observed in our case. In our patient, the adrenal mass was homogeneous, measured less than 4 cm (in the first CT and MRI), and had regular margins. Although lipid content was not typical of an adenoma, and areas of hyperintensity in T2 (possible necrosis) and T1 (possible hemorrhage) were suspicious, the lesion did not display features leading to a definite characterization. Radiologic features of adrenocortical oncocytic neoplasms are not pathognomonic (11, 15). The oncocytic variant of ACC is similar to classic adrenocortical malignant tumors, whereas oncocytomas differ from adrenocortical adenomas (from 20 to 40 Hounsfield Units and inhomogeneous enhancement versus less than 10 Hounsfield Units at CT scan) (16, 17).

Oncocytic neoplasms are usually large, surrounded by a fibrous capsule, and exhibit a non-invasive growth pattern (8). Ultrasound-guided biopsy is not carried out except in the setting of suspected adrenal metastases (7), and it cannot discriminate between renal and adrenocortical oncocytic neoplasm (18). In our patient, the lesion had a maximum diameter of 4 cm at first description, increasing to 5.5 cm after two years, when the patient was referred to our attention. ^18^F-FDG-PET showed positive uptake, but it should be borne in mind that adrenocortical oncocytomas may display false-positive uptake at ^18^F-FDG-PET, possibly due to the presence of abundant intracellular mitochondria (1). In fact, mitochondria interact with hexokinase, an enzyme responsible for the phosphorylation of glucose to glucose-6-phosphate, with consequent increase in glycolysis and trapping of ^18^F-FDG into the cells (19, 20).

On account of the increase in size and uncertainty in radiologic characterization of the lesion, it was decided to proceed with surgical removal. In principle, total adrenalectomy would have been the treatment of choice, in view of the uncertain diagnosis. The patient was informed of the risk of adrenal insufficiency following surgery, in view of previous right adrenalectomy. However, the fact that a clear cleavage plane between the lesion and the adrenal gland had been identified, allowed the selective removal of the tumor, while sparing the normal gland.

Pathologic examination revealed the lesion to be an oncocytoma. As reported in the literature, diagnosis of oncocytoma is based on morphologic and immunohistochemical features. Electron microscopy demonstrates numerous mitochondria in the tumor cell cytoplasm (21). At immunohistochemistry, neoplastic cells stain positively for inhibin (69%), melan-A (85%), synaptophysin (74%), vimentin (80%), mitochondrial antibody mES-13 (100%), calretinin (78%), neuron-specific enolase (94%), cytokeratin AE1/AE3 (52%), and negatively for chromogranin A (91%) and S100 protein (96%) (22).

In 2004, Bisceglia et al. (23) modified the Weiss system (24) to classify oncocytic adrenocortical tumors. According to the Lin-Weiss-Bisceglia diagnostic scheme, the presence of at least 1 of 3 major criteria (mitotic rate greater than 5 per 50 HPF, atypical mitoses and venous invasion) is indicative of malignancy. The presence of at least 1 of 4 minor criteria (size greater than 10 cm and/or weight greater than 200 g, microscopic necrosis, capsular invasion and sinusoidal invasion) defines an oncocytic adrenocortical neoplasm of borderline malignancy, while the absence of all criteria is indicative of a benign neoplasm. Three criteria of the original Weiss system (clear cell component less than 25%, diffuse architecture and high nuclear grade) are excluded from the Lin-Weiss-Bisceglia system and are considered as definitional for the diagnosis of an oncocytic ACC (23).

A unique characteristic of our case was that right adrenalectomy had previously been performed for Cushing syndrome due to a cortisol-secreting adenoma. Although the left adrenal gland was spared at surgery, a strict monitoring was carried out over subsequent days. While blood pressure, glycemia and serum electrolytes showed no alterations, the patient was discharged with caution and prescribed corticosteroid supplementation. Adrenocortical function was reassessed after two months and found to be normal. Cortone acetate supplementation was then discontinued, and the patient is regularly followed up at our outpatient clinic.

## Conclusion

In summary, we have described here a rare case of adrenocortical oncocytoma in a patient previously subjected to contralateral adrenalectomy for Cushing syndrome due to a cortisol-secreting adenoma. The presence of a growing adrenal mass of uncertain origin in a patient with only one adrenal gland, to our knowledge an unprecedented condition, offered a clinical challenge calling for a multidisciplinary approach. Although adrenal oncocytoma is a rare diagnosis, it should be considered in the presence of a well-demarcated, enlarging mass with non-specific radiographic characteristics.

## Data Availability

The original contributions presented in the study are included in the article/Supplementary Material, further inquiries can be directed to the corresponding author/s.
